# Uncommon *EGFR* Compound Mutations in Non-Small Cell Lung Cancer (NSCLC): A Systematic Review of Available Evidence

**DOI:** 10.3390/curroncol29010024

**Published:** 2022-01-09

**Authors:** Ilaria Attili, Antonio Passaro, Pasquale Pisapia, Umberto Malapelle, Filippo de Marinis

**Affiliations:** 1 Division of Thoracic Oncology, European Institute of Oncology, IRCCS, 20141 Milan, Italy; ilaria.attili@ieo.it (I.A.); filippo.demarinis@ieo.it (F.d.M.); 2 Department of Public Health, University of Naples Federico II, 80131 Naples, Italy; pasquale.pisapia@unina.it (P.P.); umberto.malapelle@unina.it (U.M.)

**Keywords:** uncommon, EGFR TKI, complex, double, afatinib, gefitinib, erlotinib, osimertinib, NSCLC

## Abstract

Compound epidermal growth factor receptor (*EGFR*) mutations represent a heterogeneous subgroup of non-small cell lung cancer (NSCLC) patients with uncommon *EGFR* mutations. We conducted a systematic review to investigate the available data on this patients’ subgroup. Overall, we found a high heterogeneity in the incidence of compound mutations (4–26% of total *EGFR* mutant cases), which is dependent on the different testing methods adopted and the specific mutations considered. In addition, the relative incidence of distinct compound subclasses identified is reported with extreme variability in different studies. Preclinical and clinical data, excluding *de novo* *EGFR* exon 20 p.T790M compound mutations, show good responses with EGFR tyrosine kinase inhibitors (TKIs) (combined common mutations: response rate (RR) ≥ 75% with either first- or second-generation TKIs; combined common plus uncommon: RR 40–80% and 100% with first-generation TKIs and afatinib, respectively; combined uncommon: RR 20–70%, ~80% and ~75% with first-generation TKIs, afatinib and osimertinib, respectively). Overall, data are consistent in supporting the use of EGFR TKIs in treating compound *EGFR* mutations, taking into account different sensitivity profile of accompanying *EGFR* mutations for selecting the most adequate EGFR TKI for individual patients.

## 1. Introduction

Epidermal growth factor receptor (*EGFR*) mutation-positive non-small cell lung cancer (NSCLC) identifies a molecularly selected subgroup of patients who benefit from targeted therapies. Three generations of EGFR tyrosine kinase inhibitors (TKIs)—namely gefitinib, erlotinib, afatinib, dacomitinib, osimertinib—demonstrated survival benefit over platinum-based chemotherapy and are world-wide approved in the first-line setting of advanced or metastatic *EGFR* mutant NSCLC, and a fair number of novel compounds are under investigation to prevent or overcome EGFR TKI resistance.

However, most part of such advances are related to *EGFR* exon 21 p.L858R point mutation and *EGFR* exon 19 deletions, so-called common sensitive *EGFR* mutations, overall representing about 80–90% of *EGFR* gene alterations. Approximately 10–20% of residual cases involve other mutation sites within or, even more rarely, outside the kinase domain of the receptor and are accounted as uncommon *EGFR* mutations (incidence ≤ 5% each). Evidence on the efficacy of EGFR TKIs in NSCLC patients harboring uncommon *EGFR* mutations is limited to a few prospective studies with afatinib (LUX-lung 2, 3 and 6) [1], one prospective study with osimertinib (KCSG-LU15–09) [2], and mostly retrospective series and case reports [3,4,5].

Another consistent subclass is represented by compound (also defined as complex or double or multiple) mutations. This definition includes the presence of more than one *EGFR* mutation, either common or uncommon, within the same tumor. Data specifically related to compound *EGFR* mutations are lacking. The vast majority derived from retrospective series of uncommon mutations in which the efficacy data are presented by single mutation type and not distinguished between single and compound, and often not reported. In addition, these data are highly heterogeneous, as the identification of compound mutations is dependent on the molecular testing methods adopted, often not able to properly detect the intratumor clonal heterogeneity.

The aim of this study is to systematically review the available evidence on compound *EGFR* mutations in NSCLC, with regard to prevalence, distribution and efficacy of EGFR TKI treatments, in order to provide consistent information to support treatment selection in this particularly heterogeneous subgroup of patients.

## 2. Materials and Methods

We conducted a systematic review with a PICO search according to PRISMA guidelines (Appendix A) [6]. Given that the first evidence published of EGFR TKI response in NSCLC with *EGFR* activating mutations dates back to 2004, we performed a systematic search of PubMed and Cochrane Library in the time frame between January 2004 and October 2021. The following search terms were used: “uncommon”, “EGFR”, “compound”, “complex” and “lung cancer”, with all relevant synonyms (Appendix A).

After removing duplicates, titles and abstracts were independently screened by two researchers (I.A. and A.P.).

Only English language studies published in peer-review journals were considered. Due to the infrequency of the rare condition investigated, conference abstracts were included, according to their relevance. Some unpublished studies were searched online and checked for conference abstracts retrieval from the American Society of Clinical Oncology (ASCO), the European Society of Medical Oncology (ESMO), and the American Association of Cancer Research (AACR) websites. In addition, reviews on the topic were considered for citation search. Full-text articles were read, and further selection was made based on their relevance: studies limited to single uncommon mutations, or those not reporting the proportion of compound mutations within the uncommon *EGFR* definition, were excluded. Discrepancies between the two researchers were discussed and resolved by consensus.

## 3. Results

**Figure 1:**
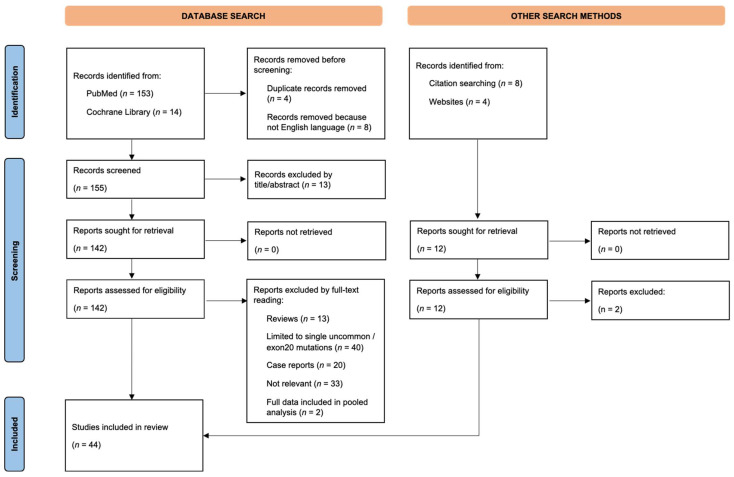
Flow diagram showing results for the systematic search performed using the search terms “uncommon”, “EGFR”, “compound”, “complex” and “lung cancer” in the time frame between January 2004 and October 2021.

The literature search yielded 153 records in PubMed and 14 records in Cochrane Library. After excluding duplicates and applying the selection criteria, 83 articles were included. An additional 7 studies were identified through cross-references/citation searching, and 4 additional conference abstracts were retrieved by website search (Figure 1). Overall, the eligible reports included 40 prospective/retrospective studies, 1 systematic review and 3 conference abstracts (Supplementary Table S1).

### 3.1. Prevalence and Distribution of EGFR Compound Mutations

Data on the prevalence of *EGFR* compound mutations in NSCLC is affected by several factors, related to ethnicity, testing methods and reporting biases.

A large amount of available evidence on testing is derived from studies conducted in Asian populations, with ~45–60% overall *EGFR* mutation rate. In these reports, the incidence of *EGFR* compound mutations ranges from 4–6.7% to 26% of *EGFR* mutant cases (Table 1) [7,8,9,10,11,12,13,14]. In Caucasian populations, three large studies have been conducted, reporting ~5–7% compound *EGFR* mutations among *EGFR*-positive patients [15,16,17]. The impact of ethnicity, and also possibly environmental factors, clearly emerges from a relevant study conducted on 2146 NSCLC in Southwest China: in the rural Qujing area, the incidence of compound *EGFR* mutations was 43.6% compared to 10.4% in the non-Qujing region (*p* < 0.0001), with patients’ occupation (farmer vs. non-farmer) being independently associated with an increased rate of *EGFR* compound mutations [12]. The incidence rate of compound mutations with respect to single *EGFR* mutations appears not to be affected by clinic-pathological features such as sex, smoking status or histology [18].

The use of different testing methods, with different limits of detection and reference range, has a significant impact on the extreme variability of compound *EGFR* rates (Table 1) and in terms of overall sensitivity as well as specificity for the different *EGFR* uncommon mutations. The largest available study on testing rate was conducted in China and included 21,324 NSCLC patients tested with either next-generation sequencing (NGS), Sanger sequencing or real-time polymerase chain reaction (qPCR) [11]. Of the 642 (6.7%) compound *EGFR* mutations identified, 71%, 49% and 35% were detected by NGS, Sanger sequencing and qPCR, respectively [11].

In addition, most of the reported studies are conducted on tumor tissue samples, either formalin-fixed paraffin-embedded (FFPE) or fresh biopsies or cytology samples. The liquid biopsy detection rate for compound *EGFR* mutations was 11% (5 out of 46 *EGFR* mutant cases as identified by liquid biopsy) in a study conducted in Indonesia with a 26% compound rate at tissue analysis [8].

As well, variability in the reporting on compound mutations accounts for biases in data interpretation. Indeed, retrospective studies focusing on treatment report a 35% compound rate among uncommon *EGFR* mutations (not fully reported reports) as identified by NGS [19,20]. In addition, some studies separately report on compound mutations, whereas some others count each mutation independently or even include the compound as a part of the representative mutation (e.g., common EGFR exon 19 deletions or EGFR exon 21 p.L858R or uncommon).

**Figure 2:**
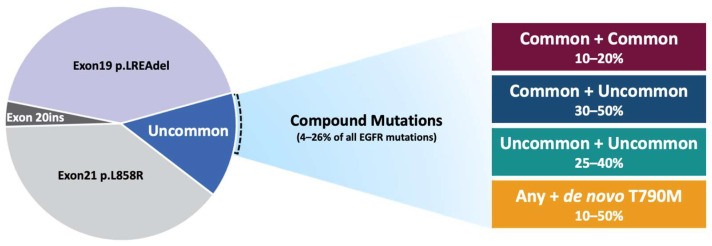
Pie chart representing the EGFR mutation landscape. Compound mutations account for about 4–26% of all *EGFR* mutations. This heterogeneous group comprises: common + common (e.g., exon 21 p.L858R + exon 19 deletions); common + uncommon (e.g., exon 21 p.L858R + exon 20 p.S768I); uncommon + uncommon (e.g., exon 18 p.E709A + exon 18 p.G719C); any + de novo exon 20 p.T790M (e.g., exon 21 p. L858R + exon 20 p.T790M).

This is particularly relevant when addressing the issue of the distribution of compound *EGFR* mutations. For this purpose, according to the available data on testing and outcomes, we identified four main categories of compound *EGFR* mutations: combined common *EGFR* mutations (exon 21 p.L858R + exon 19 deletions), combined common (exon 21 p.L858R + exon 19 deletions) plus uncommon *EGFR* mutations (any but exon 21 p.L858R, exon 19 deletions or *de novo* exon 20 p.T790M), combined uncommon *EGFR* mutations and combined *EGFR* mutation (any) plus *de novo* exon 20 p.T790M (Figure 2). Triple or more compound mutations are categorized within the four subgroups according to the presence or absence of uncommon or *de novo* exon 20 p.T790M mutations. Additionally, despite the known negative prognostic and predictive role with EGFR TKIs of *EGFR* exon 20 insertions [21,22,23], these mutations are accounted as uncommon mutations and not as separate group because of not consistent systematic reporting on compound mutations in this specific subgroup.

As previously stated for the overall compound *EGFR* mutation rate, the use of testing methods with different sensitivity and specificity, and the reporting biases, justifies the huge variability of the four subgroups’ distribution. Combined common *EGFR* mutations are not reported in some studies, ranging from ~10–20% where reported. The rate of combined common plus uncommon mutations (~30–50%) is similar to that of combined uncommon (~25–40%), both in Asian and non-Asian populations [7,9,11,12,13] (Figure 2). The most frequent uncommon mutations detected in the compound *EGFR* mutations are represented by the major uncommon exon 18 p.G719X, exon 20 p.S768I and exon 21 p.L861Q. Conversely, the rate of *de novo* exon 20 p.T790M compound *EGFR* mutations (~10–50%) appears to be affected by different testing methods. In particular, among the 261 (40.7%) *de novo* exon 20 p.T790M compound mutations identified within the largest Chinese cohort [11], only 57 (21.9%) were detected by NGS, whereas 135 (51.7%) by qPCR and 69 (26.4%) by Sanger sequencing.

### 3.2. Preclinical Data on EGFR Compound Mutations

Evidence from preclinical studies outline that the efficacy of EGFR TKIs on compound *EGFR* mutation is significantly affected by the sensitivity pattern of the accompanying *EGFR* mutations [24]. As an example, in vitro experiments showed reduced responses to gefitinib in double mutants exon 18 p.E709A + exon 18 p.G719C, exon 20 p.Q787R + exon 21 p.L858R and exon 21 p.H870R + exon 21 p.L858R compared with exon 18 p.G719C or exon 21 p.L858R alone [24]. Similarly, in vitro erlotinib efficacy appears to be concentration dependent in double exon 18 p.G719A/S + exon 21 p.L861Q [25]. Conversely, afatinib revealed stronger inhibitory profile against a wide spectrum of uncommon mutations [25,26,27,28]. The third-generation EGFR TKI osimertinib also showed in vitro activity, though less markedly, against compound *EGFR* mutations (45 out of 69 compound mutations were highly sensitive to osimertinib, compared to 62 highly sensitive to afatinib) [28], and this was confirmed also in PDX-models of double mutants exon 18 p.G719A + exon 20 p.S768I, exon 18 p.G719C + exon 20 p.S768I and exon 18 p.G719A + exon 21 p.L861Q [27].

As a matter of fact, the presence of *de novo* exon 20 p.T790M in combination with any other *EGFR* mutations confers the higher grade of resistance to first- and second-generation EGFR TKIs [24,29].

### 3.3. Clinical Outcomes of NSCLC Patients Harboring EGFR Compound Mutations

Evidence on the efficacy of different EGFR TKIs is markedly heterogeneous and mostly derives from retrospective studies.

Overall, available literature supports the use of EGFR TKIs as first-line treatment of advanced or metastatic NSCLC patients harboring *EGFR* compound mutations [30]. The median overall survival (OS) appears longer in NSCLC patients with compound mutations than in those with single uncommon *EGFR* mutations, consistently across different retrospective studies (~31–33 vs. 12–17 months) [31,32,33,34,35].

#### 3.3.1. First-Generation EGFR-TKIs in Compound EGFR Mutations

In a retrospective study on 99 patients with uncommon *EGFR* mutations, those with compound mutations had longer progression-free survival (PFS) and OS with first-generation EGFR-TKIs as compared to first-line chemotherapy (PFS 9.3 vs. 5.3 months; OS 31.4 vs. 16.8 months, respectively) [31]. Data supporting the use of first-generation EGFR TKIs are stronger when treating combined common or common plus uncommon *EGFR* mutations [33,36,37,38]. In a small retrospective study focusing on exon 21 p.L858R mutations, there was no difference in response and survival with gefitinib among single and compound exon 21 p.L858R groups [39]. Similar results were shown in another small study, demonstrating no significant differences with gefitinib in response rate (RR), PFS and OS in compound mutations with a common *EGFR* mutation with respect to single common *EGFR* mutations (RR 83% vs. 73%, *p* = 0.52; PFS 12.7 vs. 8.1 months, *p* = 0.39; OS 24.7 vs. 16.1 months, *p* = 0.170) [40]. Another small study reported on 16 patients with compound mutations treated with gefitinib: RR was 86% in combined common vs. 40% in combined common plus uncommon mutations [41]. In addition, first-generation EGFR TKIs also reported good response rates in treating combined major uncommon *EGFR* mutations. RR was 86% in a small report on 11 compound *EGFR* patients treated with erlotinib, including combined common plus uncommon and combined major uncommon mutations [42]. In another retrospective study, RR and PFS were higher in combined major uncommon exon 18 p.G719 + exon 21 p.L861 (*n* = 28 out of 62) compared to other combined mutations (RR 57.1% vs. 20%, PFS 6 vs. 1.6 months) [43].

Of note, in a retrospective study excluding *de novo* exon 20 p.T790M cases, patients with combined common plus uncommon mutations were reported to have significantly better outcomes with gefitinib compared to compound without common *EGFR* accompanying mutations (RR 83% vs. 29%, *p* = 0.045; PFS 12.7 vs. 4.9 months, *p* = 0.048; OS 24.7 vs. 12.3 months, *p* = 0.027) [40]. Consistently, in a large retrospective study (*n* = 187) with first-line first-generation EGFR TKIs in 51 patients harboring *EGFR* compound mutations, RR was 75% in the combined common, 60% in combined common plus uncommon and 71% in combined uncommon subgroups [44]. Median PFS were 18.2, 9.7 and 9.6 months, respectively [44].

**Figure 3:**
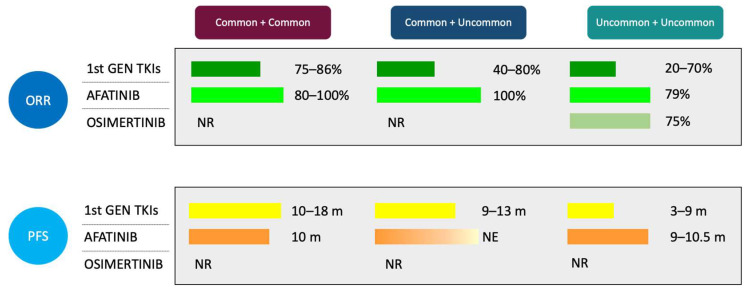
Clinical outcomes of compound *EGFR* mutations with different EGFR TKIs; *de novo* T790M compound mutations are excluded. NR: not reported. NE: not estimable.

Another retrospective study included 102 NSCLC patients with *EGFR* uncommon mutations and 99 with single *EGFR* mutations as control group, treated with first- or second-generation EGFR TKIs as first-line treatment [45]. Of note, among patients treated with first-line EGFR TKIs, RRs in combined common plus uncommon and combined uncommon *EGFR* mutations were lower compared to single common *EGFR* mutations (objective response rate, ORR 54.5% and 44.4% vs. 75%, respectively). In addition, RRs in combined uncommon and combined common plus uncommon were higher as compared to single uncommon *EGFR* mutations (ORR 44.4% and 54.5% vs. 21.4%, respectively). No differences were observed among compound subgroups treated with second-generation afatinib. Moreover, afatinib showed higher ORR and longer PFS compared to first-generation TKIs in combined common plus uncommon mutations (ORR 100% vs. 54.5%, *p* = 0.017; PFS NE vs. 13.6 months, *p* = 0.032, respectively) [45] (Figure 3).

#### 3.3.2. Second-Generation EGFR-TKIs in Compound EGFR Mutations

Available data on afatinib are more solid and include evidence deriving from prospective studies. The afatinib uncommon mutations database included 40 NSCLC patients with compound mutations [46]. In this subgroup, RR was 77% and median time to treatment failure was 14.7 months, reaching 16.6 months in those with at least one major uncommon mutation. Of note, responses were also observed with afatinib in the EGFR-TKI pretreated setting (ORR 28.6%) [46].

In a retrospective study focusing on compound mutations, 125 NSCLC patients with these alterations, excluding *de novo* exon 20 p.T790M compounds, received an EGFR TKI as first-line treatment [47]. Overall, treatment with afatinib showed longer PFS than gefitinib and erlotinib, and longer OS than erlotinib. In cases of compound common *EGFR* mutations, no difference was observed among the three drugs in terms of response (RR: gefitinib 83%, erlotinib 73.7%, afatinib 88.2%) and PFS (gefitinib 10.9, erlotinib 8.5, afatinib 9.6 months, *p* = 0.385). Conversely, afatinib demonstrated higher RR and prolonged PFS in those patients with combined uncommon pattern (RR: afatinib 78.9%, gefitinib 38.9%, erlotinib 20%, *p* = 0.013; PFS: afatinib 10.5, gefitinib 3, erlotinib 0.9 months) [47] (Figure 3).

Conversely, data on the activity of dacomitinib on compound *EGFR* mutations are scant: despite initial signals of activity in vitro on acquired compound mutations after osimertinib resistance, no clinical activity was confirmed in this setting, so far [48].

#### 3.3.3. Third-Generation EGFR TKIs in Compound EGFR Mutations

A small phase 2 prospective trial of osimertinib in uncommon *EGFR* mutations (KCSG-LU15–09) enrolled four patients with compound mutations (two double mutants exon 18 p.G719X + exon 20 p.S768I and two with exon 18 p.G719X + exon 21 p.L861Q) in Asian populations [2]. Response was obtained in three of the four patients.

A retrospective cohort study conducted in the US, median time on osimertinib in 14 patients with major uncommon *EGFR* mutations (including 10 compound cases) was 8.9 months. Of note, patients with exon 21 p.L861Q (5 out of 6 cases as compound mutations) had longer time to treatment failure as compared to exon 18 p.G719X cases (7 out of 10 as compound mutations): 19.3 vs. 5.8 months, *p* = 0.008 [49] (Figure 3).

#### 3.3.4. Exon 20 p.T790M EGFR Compound Mutations

Consistently with data reported in the preclinical setting, the co-occurrence of *de novo* exon 20 p.T790M *EGFR* mutation with any other *EGFR* mutation confers primary resistance to first- and second-generation EGFR TKIs and is associated with shorter PFS [50]. Indeed, when *de novo* exon 20 p.T790M compound *EGFR* mutations are accounted together with all the compound *EGFR* mutations, no difference in response rate was observed between the use of first-generation TKIs and chemotherapy (ORR 47% vs. 43.4%, respectively). Conversely, non-exon 20 p.T790M uncommon/compound mutations obtained benefit from the use of TKIs as compared to chemotherapy (ORR 80% vs. 57%), whereas *de novo* exon 20 p.T790M patients reported ORR 11% with EGFR TKIs and 27% with chemotherapy [51]. Another retrospective study reported ORR 8.3% and median PFS 1.4 months in combined *de novo* exon 20 p.T790M mutant NSCLC patients receiving first-line treatment with first-generation EGFR TKIs [44].

Conversely, responses were observed in nine *de novo* exon 20 p.T790M patients treated with osimertinib (RR 33.3%, DCR 100%), including five *de novo* exon 20 p.T790M compound mutations [19].

Different is the scenario of acquired exon 20 p.T790M resistance mutation in patients with *EGFR* compound mutations. Limited data are available in this setting, consistent with a reduced response (RR 27%) and survival to osimertinib administration at the occurrence of resistance when compared to patients with single *EGFR* mutations and acquired exon 20 p.T790M (median PFS 2.9 vs. 9.7 months; median OS 17.8 vs. 31 months) [52].

## 4. Discussion

Compound *EGFR* mutations represent a highly heterogeneous subgroup of uncommon *EGFR*-positive NSCLC patients. The specificity of the accompanying mutations accounts for the huge variability in response and survival with different generations of EGFR TKIs.

In this manuscript we systematically reviewed the available literature focusing on compound *EGFR* mutations. The bulk of the evidence is derived from retrospective studies, most of them limited in sample size due to the rarity of the condition investigated. In addition, most studies include the compound mutations as an individual subgroup of uncommon *EGFR* mutations and only few of them report differential efficacy among distinctive compound subclasses.

According to the reviewed data on relative incidence and clinical outcomes, we were able to classify four compound subgroups: two with common mutational pattern (combined common and combined common plus uncommon *EGFR* mutations) and overall similar responses and survival outcomes with any EGFR TKIs as compared to patients with single common *EGFR* mutations; one subgroup with uncommon mutational pattern (combined uncommon *EGFR* mutations), reporting higher benefit with second- and third- generation TKIs compared to gefitinib or erlotinib data; and finally, *de novo* exon 20 p.T790M compound *EGFR* subgroup, characterized by poor responses and worse prognosis.

Testing emerged as one of the crucial points in addressing the issue of compound *EGFR* mutations. Different techniques are currently adopted in molecular predictive pathology laboratories for molecular purposes (Table 2). However, due to wide reference range, the higher multiplexing power, the low costs, the limited TAT and the possibility to optimize tissue or liquid biopsy samples for the different molecular biomarkers, NGS approaches should be preferred to single gene testing [PMID: 34813925].

The widespread of NGS platforms allows for the identification of increasing number of rare *EGFR* mutations, which often occur as compound. This provides a more reliable snapshot of intra-tumoral heterogeneity within *EGFR*-positive NSCLC patients, in terms of diagnosis but also for the prediction of clinical outcomes.

In this view, the use of such novel testing methods is highly recommended in order to adequately detect specific aminoacidic substitutions in accompanying rare uncommon mutations (e.g., exon 18 p.G719A/D/S and not simply exon 18 p.G719X) and to provide evidence on different spectrum of response to EGFR TKIs for each specific mutation subtype to guide treatment selection. In the absence of consistent prospective data, sensitivity of constituent mutations should be always considered for the tailored treatment selection of patients harboring compound *EGFR* mutations.

## Figures and Tables

**Table 1:** Main studies reporting the testing rate of *EGFR* compound mutation in NSCLC tissue samples.

Study	Region	Patients Screened (N)	Testing Method	*EGFR* Mut Rate	*EGFR* Compound Mut Rate
				(N, %)	(N, % of *EGFR* Mut)
Syahruddin et al., 2018 [7]	Indonesian	1779	PCR HRM	791 (44.4)	154 (19.5)
			RFLP		
Zaini et al., 2019 [8]	Indonesian	116	PCR HRM	69 (63.2)	18 (26)
			RFLP		
Jing et al., 2018 [9]	China	112	NGS	58 (51.8)	11 (18.9)
Mao et al., 2021 [11]	China	21,324	NGS + qPCR + Sanger	9,621 (47.5)	642 (6.7)
Wen et al., 2019 [14]	China	1200	NGS	571(47.6)	87 (15.3)
Zhou et al., 2021 [12]	SW China	2146	ARMS-PCR	346 (46) Q	151 (43.6) Q
	(Q vs. non-Q)			710 (51) non-Q	74 (10.4) non-Q
Namba et al., 2019 [10]	Japan	531	MBS	64 (n.e.) ^1^	8 (12.5)
Shi et al., 2013 [13]	Malaysia	484	ARMS + HRM	221 (45.7)	9 (4)
Evans et al., 2019 [15]	EU	17,782	qPCR	1,737 (10.7)	79 (4.9)
Sousa et al., 2020 [17]	EU	1228	Sanger	252 (20.5)	19 (7.5)
Martin et al., 2019 [16]	EU	2906	Sanger	408 (14)	22 (5.4)

Not evaluable: randomly selected. ARMS: amplification refractory mutation system; EU: Europe; HRM: high resolution melt; MBS: amplicon-based targeted sequencing with the molecular barcoding system; NGS: next-generation sequencing; PCR: polymerase chain reaction; Q vs. non-Q: Qujing City vs. non-Qujing City; qPCR: real-time PCR; RFLP: restriction fragment length polymorphism.

**Table 2:** Advantages and disadvantages of the main molecular techniques.

Methodology	Advantages	Disadvantages
RT-PCR	- rapid (low TAT) - low costs - extensively adopted in molecular predictive pathology laboratories	- low limit of detection - ability to detect only known and well characterized alterations - limited multiplexing power
dPCR	- rapid (low TAT) - low costs - possibility to detect variant at low allelic frequency (high sensitivity)	- detection of only known and well characterized alterations - limited multiplexing power
NGS	- possibility to detect variant at low allelic frequency (high sensitivity) - ability to detect all variant within the gene panel adopted (broad reference range) - multiplexing power	- careful validation, in particular for non-FFPE samples - bioinformaticians support is required - high specialized and trained personnel

Abbreviations: cfNAs: circulating free nucleic acids; dPCR: digital polymerase chain reaction; FFPE: formalin-fixed paraffin-embedded; NGS: next-generation sequencing; RT-PCR: real-time polymerase chain reaction; TAT: turnaround time.

## Data Availability

Not applicable.

## Supplementary Materials

The following are available online at https://www.mdpi.com/article/10.3390/curroncol29010024/s1, Table S1: studies included in review.

## Appendix A

Systematic PICO search:

(“EGFR” OR “epidermal growth factor receptor”) AND (“compound mutations” or “complex mutations” or “uncommon mutations”) AND (“lung cancer” OR “lung neoplasms”).
