# Supplementary material for: Uncommon EGFR Compound Mutations in Non-Small Cell Lung Cancer (NSCLC): A Systematic Review of Available Evidence
Source: Curr Oncol. 2022 Jan 9;29(1):255–66. doi: 10.3390/curroncol29010024 (PMC8774526; doi:10.3390/curroncol29010024)
Supplement: Supplementary file 1 [file curroncol-29-00024-s001.zip › curroncol-1486520-supplementary.pdf]

Supplementary Table S1. Details of the studies included in the systematic review

**Studies included**	**Study type**
**Cho, J.H., et al., J Clin Oncol, 2020.**	Prospective trial
**Yang, J.C., et al., Lancet Oncol, 2015.**	Pooled analysis of prospective studies
**Passaro, A., et al., Front Oncol, 2021.**	Pooled analysis of prospective studies
**Syahruddin, E., et al., Lung Cancer, 2018.**	Retrospective study
**Zaini, J., et al., Cancer Rep, 2019.**	Prospective study
**Jing, C., et al., Molecular medicine reports, 2018.**	Translational study
**Namba, K., et al., BMC Cancer, 2019.**	Retrospective study
**Mao, L., et al., Pathol Oncol Res, 2021.**	Retrospective study
**Zhou, Y., et al., Frontiers in Oncology, 2021.**	Retrospective study
**Shi Yeen, T.N., et al., Journal of biomedical science, 2013.**	Translational study
**Wen, S., et al., Oncologist, 2019.**	Translational study
**Evans, M., et al., Pathol Oncol Res, 2019.**	Retrospective study
**Martin, J., et al., Clin Lung Cancer, 2019.**	Retrospective study
**Sousa, A.C., et al., Lung Cancer, 2020.**	Retrospective study
**Hayashi, T., et al., Hum Pathol, 2020.**	Retrospective study
**Tam, I.Y., et al., Mol Cancer Ther, 2009.**	Preclinical study
**Kimura, S., et al., Cancer science, 2018.**	Preclinical study
**Gristina, V., et al., Cancer Treat Rev, 2020.**	Systematic review
**Floc'h, N., et al., Mol Cancer Ther, 2020.**	Preclinical study
**Kohsaka, S., et al., Sci Transl Med, 2017.**	Preclinical + retrospective study
**Akula, S., et al., Journal of Thoracic Oncology, 2018.**	Preclinical study
**Peng, L., Z.-G. Song, and S.-C. Jiao, Scientific Reports, 2014.**	Retrospective study
**Li, H., et al., Lung Cancer, 2019.**	Retrospective study
**Rossi, S., et al., Curr Probl Cancer, 2021.**	Retrospective study
**Passaro, A., et al., Clin Lung Cancer, 2019.**	Retrospective study
**Moran, T., et al., Clin Lung Cancer, 2020.**	Retrospective study
**Chen, K., et al., Cancer Chemother Pharmacol, 2017.**	Retrospective study
**Lei, L., et al., Cancer Med, 2020.**	Retrospective study
**Xu, J., et al., Lung Cancer, 2016.**	Retrospective study
**Jung, H.A., et al., Biology (Basel), 2020.**	Retrospective study
**Peng, L., Z. Song, and S. Jiao, Onco Targets Ther, 2015**	Retrospective study
**Wu, S.G., et al., Oncologist, 2008.**	Retrospective study
**Hata, A., et al., J Thorac Oncol, 2010.**	Retrospective study
**Kobayashi, S., et al., J Thorac Oncol, 2013.**	Retrospective study
**Wu, J.Y., et al., Clin Cancer Res, 2011.**	Retrospective study
**Zhang, B., et al., Cancer, 2018.**	Retrospective study
**Tan, J., et al., Front Oncol, 2021.**	Retrospective study
**Yang, J.C.-H., et al., Journal of Thoracic Oncology, 2020.**	Pooled analysis of prospective/retrospective/case report studies
**Wu, S.G., et al., Ther Adv Med Oncol, 2020.**	Retrospective study
**Singh, V., et al., Lung Cancer, 2020.**	Combined retrospective/prospective
**Lin, Y.T., et al., Lung Cancer, 2020.**	Retrospective study
**Bar, J., et al., 1206P Annals of Oncology, 2021.**	Conference abstract
**Miura, S., et al., Abstract LB138: Cancer Research, 2021.**	Conference abstract
**Ji, J., et al., Journal of Clinical Oncology, 2020.**	Conference abstract

N.R.: not reported
